# Investigating Unused Tools for the Animal Behavioral Diversity Toolkit

**DOI:** 10.3390/ani12212984

**Published:** 2022-10-30

**Authors:** James Edward Brereton, Eduardo J. Fernandez

**Affiliations:** 1 Animal and Zoo Science, University Centre Sparsholt, Westley Lane, Sparsholt, Winchester SO21 2NF, UK; 2 School of Animal and Veterinary Sciences, The University of Adelaide, Adelaide, SA 5005, Australia

**Keywords:** animal welfare, behavioral diversity, behavioral variability index, chao, Shannon–Wiener index, Simpson’s index

## Abstract

**Simple Summary:**

Behavioral diversity is sometimes used by animal scientists to better understand and compare how animals behave. The methods used in behavioral diversity research have not been investigated across the entire research sector. This paper aims to identify which methods are being used in behavioral diversity research and find some novel methods from other areas of science that could be used in new research. To investigate the techniques and species investigated in behavioral diversity literature, a literature search was conducted. Two methods: behavioral richness and the Shannon–Wiener index, were the most often used methods, whereas the Behavioral Variability index featured rarely. While a range of species appeared in the behavioral literature, mammals were the most frequently studied animal group, whereas amphibians did not feature in any papers. There are several diversity indices which did not feature in behavioral diversity including Simpson’s index, and Chao. These methods could be used to better understand animal behavioral study outputs or be used to estimate the number of ‘unobserved’ behaviors that an animal may express. Future studies could therefore make use of these unused tools.

**Abstract:**

Behavioral diversity is a commonly used tool used to quantify the richness and evenness of animal behaviors and assess the effect of variables that may impact an animal’s quality of life. The indices used in behavioral diversity research, and the study subjects, have not been formally reviewed. This paper aims to identify which indices are being used in behavioral diversity research, and under which scenarios, and uncover novel indices from other disciplines that could be applied to behavioral diversity. To investigate the techniques and species investigated in behavioral diversity literature, a Web of Science literature search was conducted. Two methods: behavioral richness and the Shannon–Wiener index, were the most frequently used indices, whereas the Behavioral Variability index featured rarely. While a range of species appeared in the behavioral literature, mammals were the most frequently studied Class, whereas amphibians did not feature in any papers. There are several diversity indices which did not feature in behavioral diversity including Simpson’s index, and Chao. Such indices could be used to better understand animal behavioral study outputs or be used to estimate the number of ‘unobserved’ behaviors that an animal may express. Future studies could therefore extend beyond the Shannon–Wiener and richness indices.

## 1. Introduction

Behavioral diversity indices are frequently used in animal behavioral research [1]. The indices have been applied to animals housed in a wide array of environments, including the wild [2], zoos [3], kennels [3] and farms [4,5]. For the purpose of this paper, behavioral diversity is defined as any index that is used to quantify the variety of behaviors or behavior categories that an animal expresses during a study period. The term behavioral ‘richness’ is defined as the number of behavior categories observed, and ‘evenness’ is defined as the variance in quantity of behavior between behavior categories (see Supplementary Table Of Key Terms, Table A1) [1].

While also used for other research types, behavioral diversity is sometimes used to measure animal welfare. The theory here is that enriched animals have the opportunity to engage in a much wider range of behaviors that those in impoverished environments. Similarly, an animal that is experiencing poor welfare due to chronic pain or stress may choose to remain inactive for long periods, even when opportunities are available. Some animals in similar positions engage in stereotypy; long bouts of repetitive, functionless behavior that also result in low diversity scores on account of their invariant nature [6,7]. An animal, therefore, that engages in only a few behaviors is purported to have a poor welfare score according to behavioral diversity theory [8,9,10]. Improvements to the environment or pain scale may increase the range of behaviors that the animal engages in, thereby improving their environmental ‘choice and control’ [11].

Several cautionary notes have arisen regarding the application of behavioral diversity to animal studies [12]. There are issues, for example, around the frequency and type of behavioral observations and the categorization of behavior (i.e., a highly detailed or vague ethogram), both of which will influence the values generated and thus the interpretation of the results. Similarly, consideration must be paid to the ecological relevance of the findings, as many species naturally spend long periods engaged in ‘sit and wait’ foraging activities, and poor behavioral diversity thus does not always demonstrate poor welfare. Similarly, an animal that expresses multiple behaviors with negative welfare connotations (e.g., self-directed) would score highly in behavioral diversity. It is beyond the scope of this paper to highlight all key limitations: readers are encouraged to read Cronin and Ross [12] if planning behavioral diversity projects. It is similarly beyond the scope of this paper to determine whether greater behavioral diversity equates to good welfare, and this should be reviewed on an individual scenario basis, with consideration of the actual behaviors that are observed.

Behavioral diversity is a measure in that it can be applied alongside other welfare tools. In addition to behavior, this measure has been previously applied alongside other welfare-related metrics such as enclosure use indices [12,13,14] or assessment of glucocorticoid metabolites [10,15].

Behavioral diversity has potential as an objective comparative measure in carefully planned behavior studies. Behavioral diversity could be used to measure the impact of an intervention such as an enclosure move, novel environmental enrichment, or training effectiveness [16,17,18,19]. While a couple of techniques have been developed specifically for captive animal research, such as the Behavioral Variety Index [20,21], most behavioral diversity indices have been adapted from other fields, such as mathematics [22] and ecology [23]. Thus, some indices have already been used for decades in the guise of ‘species diversity’ indices for ecological biodiversity sampling studies, or studies of animal microbiomes [24]. Studies in ecology and microbiology have developed strategies to cope with challenges such as animals in a habitat that are yet to be observed and behavioral evenness [22]: these problems are not always tackled in the behavioral diversity literature.

While an excellent review of behavioral diversity literature is available in the literature [1], there is a need to investigate which indices are most commonly used, and whether there is scope to extend the range of tools available. For instance, although behavioral diversity studies make good use of several indices, such as the Shannon–Wiener index [22,25,26], there remain many indices that have not yet been used by behavioral biologists. Additionally, it is important to identify where and how behavioral diversity indices are currently being applied to a range of different species. The purpose of this study is to quantify the existing behavioral diversity literature and identify new tools that could add value to future research.

## 2. Behavioral Diversity: Literature Review and Categories

Relevant papers were identified using a search of the Web of Science *™* database. The search period was set for a 71-year window (from 1 January 1951 to 31 December 2021). The search terms used were “behavioral diversity” or “behavioural diversity” followed by “animal”. Papers were counted if they were original research papers and had behavioral diversity outputs for named animal species. Papers were excluded if they were reviews, did not state the species under investigation, or did not provide any behavioral diversity outputs. Papers that did not investigate living organisms explicitly (e.g., papers investigating behavioral diversity of robots) were similarly excluded. For all included papers, the species being studied and behavioral diversity index that was used was recorded.

To identify other potential diversity indices, an ad hoc sample of studies from ecology and microbiology were identified, because both fields commonly investigate the diversity of species in their respective areas. Papers were identified by using the search term ‘species diversity’ plus ‘microbiome’ or ‘ecology’ using the database and timeframes detailed above. A convenience sample of papers were collected from these searches, but it was beyond the scope of this paper to summarize all papers. The species diversity indices from these papers were noted along with their equations.

## 3. Summary of Behavioral Diversity Literature

A total of 52 papers were identified from the literature search, with a total of 112 species investigated in these papers. Here, the term ‘paper’ is used to describe a published manuscript, whereas the term ‘study’ is used to define specific experiments/observations. Therefore, a paper could contain more than one study, particularly on occasions where multiple species were observed. The most common method of behavioral diversity assessment was the Shannon–Wiener index with 79 individual species studied using this method, with behavioral richness (the number of behaviors observed) also appearing commonly (31 species studied) (Table 1). The Behavioral Variety index (proportion of behaviors seen in captive, versus wild animals) featured in two papers [20,21].

## 4. Tools to Measure Behavioral Diversity

While the Shannon–Wiener index and behavioral richness are regularly used, many potential indices have not yet been applied to behavioral diversity studies. These potential tools include Simpson’s, Menhinick’s and Chao indices [24]. These tools may have synergistic value alongside existing indices, allowing a researcher to estimate the true behavioral diversity of an animal or take behavioral evenness into consideration.

### 4.1. Behavioral Richness

Behavioral richness is the simplest measure available. Behavioral richness is defined as a measure of the number of behaviors observed during a set period [27]. For example, if an animal shows eight categories of behavior during a study, the behavioral richness value is eight. This allows for rapid comparison between study subjects or conditions, and the numbers generated are understandable (they are a count of categories of behavior seen) [28]. While simple, there remain three major limitations to the use of behavioral richness: (1) evenness in behaviors observed, (2) sampling effort and (3) ethogram access.

As behavioral richness considers only the number of behaviors seen, it does not take into account the evenness with which these behaviors occur. For example, animals will receive identical scores if they perform the same number of behaviors, regardless of how evenly they perform them [29].

Another challenge faced by behavioral richness is it cannot control for sampling effort. This challenge emerges in situations where one animal is observed more frequently than another (e.g., because one animal spends long periods out of sight). Here, the researcher is intrinsically more likely to identify higher behavioral richness to the visible animal, despite the fact that the two may actually be similar in behavioral diversity [30]. While this issue can in part be reduced by good experimental design (e.g., consideration of interval length), issues remain, especially where animals have access to out-of-sight areas [26]. Fortunately, alternative tools are available that take both evenness and sampling effort into account (see the following sections).

There are also challenges in that detailed ethograms are not always available, even for some commonly housed species. To address this, further basic behavioral science is needed to better standardize how behaviors are measured. Simply stated, richness will vary based on the use of different ethograms or behavior types observed, which makes interpreting different richness results across different settings and studies difficult.

### 4.2. Shannon–Wiener Index

The Shannon–Wiener index appears to be the most commonly used behavioral diversity index [11,31,32]. While simple, the index takes into consideration the evenness of each behavior, avoiding issues raised with behavioral richness. The equation for the Shannon–Wiener index is:

$$H^{\prime }=-\overset{S}{\underset{i=1}{\sum }}p_{i}Lnp_{i}$$

**Figure 1:**
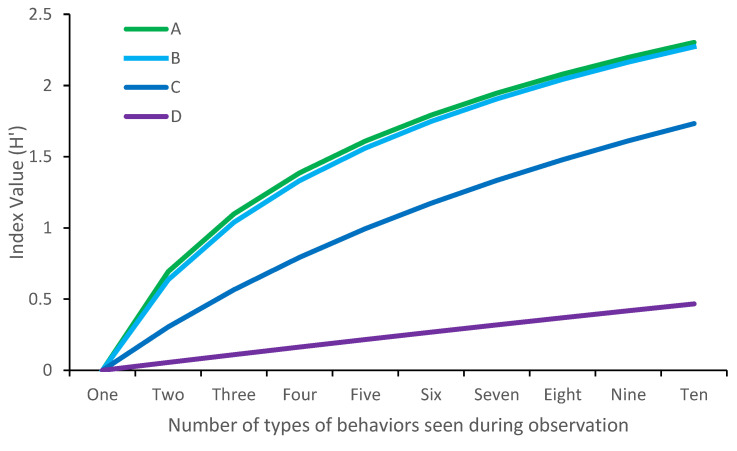
The effect of number of behaviors seen on Shannon–Wiener index outputs, using simulated data. Simulation A demonstrates H’ outputs when all behaviors are seen an equal number of times. Simulation B, C and D demonstrate H’ outputs when all behaviors occur equally frequently, apart from the first behavior which occurs 2×, 10× or 100×, respectively, more frequently than the others.

Here, *p_i_* refers to the proportion of time the animal spent engaged in the *i*th behavior [22]. Shannon indices range from 0 (only one behavior is seen) to infinite, and higher scores are provided to observations with greater behavioral richness and high evenness. There is a maximum *H′* value per number of behaviors seen: this occurs when all behaviors are equally represented (Figure 1).

The main benefit to the Shannon–Wiener index over behavioral richness, therefore, is that evenness is considered. A single value accounting for both evenness and richness has great application in terms of quantifying behavioral change or differences between individual animals [32,33]. The values generated from this index are not always intuitive, however. For example, a H’ index of 2.30 may not mean much, except to those familiar with the index. This index is also slightly less sensitive to differences in sample size (e.g., as a result of out of sight animals) as the index is generated based on proportional data rather than raw counts [34]. Finally, it is worth pointing out that the Shannon–Wiener index has also been described as Entropy, which has similarly been used to assess enclosure use variability [13,35,36].

### 4.3. Simpson’s Index

Despite their wide application across the field of ecology [30], Simpson’s index rarely features in behavioral diversity papers. There are actually three different indices which fall under the umbrella of ‘Simpson’s index. The equation for the simplest, known as Simpson’s index, or D, is as follows:

$$D^{\prime }=\overset{k}{\underset{i=1}{\sum }}\left(\frac{n_{i}(n_{i}-1}{n\left(n-1\right)}\right)$$

**Figure 2:**
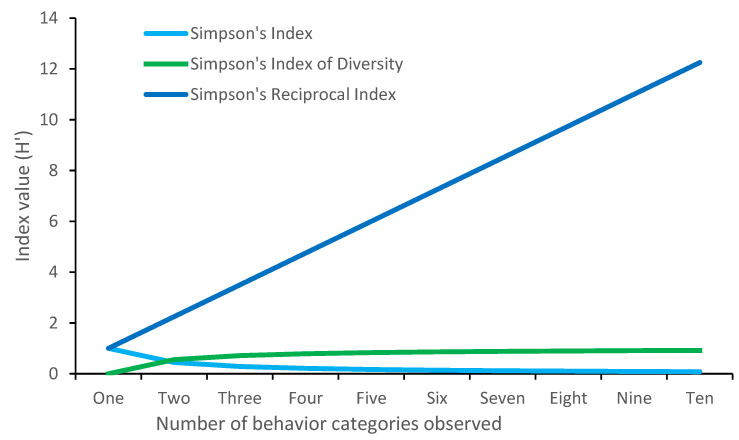
Comparison of Simpson’s index, Simpson’s index of diversity, and the Simpson’s Reciprocal index, using an identical dataset where all behaviors are equally well represented.

Here, *n* refers to the total number of observations made and *n_i_* refers to the number of observations of the *i*th behavior. The values of Simpson’s index vary between 1 (lowest diversity) and 0 (highest diversity). Many researchers find these numbers counterintuitive, with the lowest diversity resulting in the highest value. As such, the second index, Simpson’s index of Diversity, was produced. This index can be calculated by simply deducting the Simpson’s index from 1, (1—Simpson’s index), and results in inverted values, where 1 demonstrates highest diversity and 0 the lowest. The final index, Simpson’s Reciprocal index, can be calculated as 1/Simpson’s index, where 1 is the lowest value and the upper limit is infinite (Figure 2).

**Figure 3:**
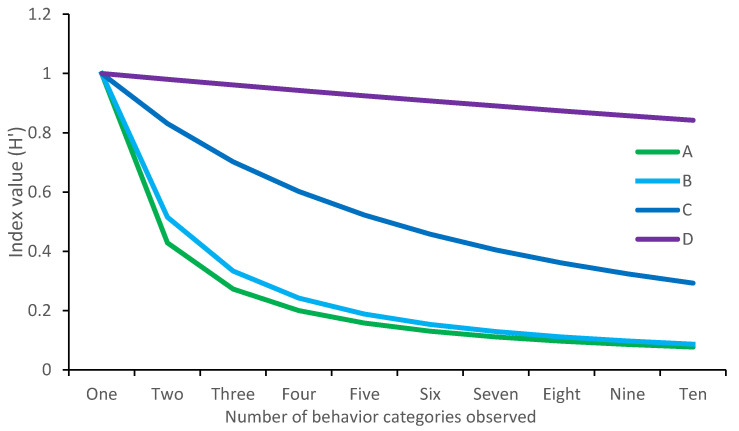
The effect of number of behaviors seen on Simpson’s index outputs, using simulated data. Simulation A demonstrates H’ outputs when all behaviors are seen an equal number of times. Simulation B, C and D demonstrate H’ outputs when all behaviors occur equally frequently, apart from the first behavior which occurs 2×, 10× or 100×, respectively, more frequently than the others.

Much like the Shannon–Wiener index, Simpson’s index is sensitive to both behavioral richness and evenness [37]. However, the outputs of Simpson’s index are more structured, with a highest possible behavioral diversity value of 0 (or 1 for Simpson’s index of diversity; DeJong 1975). This means that large differences result in comparatively smaller changes in index values, in comparison to the Shannon–Wiener index. Only large-scale changes, for example in behavioral evenness, will result in noticeable index changes (Figure 3). Statistical tests (e.g., *t* tests) could be used to investigate differences in index values between conditions, but this depends on the questions being asked alongside test assumptions.

**Figure 4:**
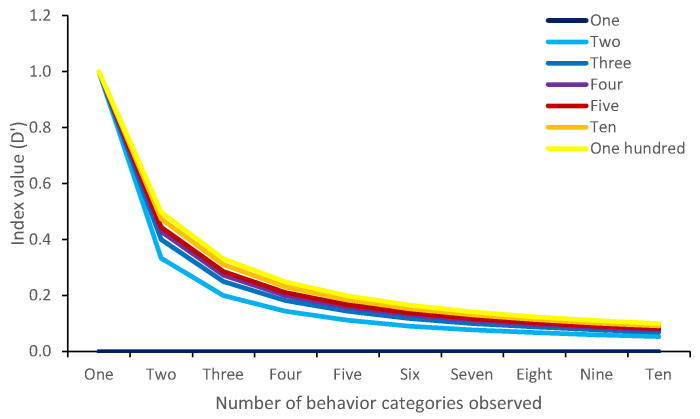
The effect of the number of observations on the Simpson’s index values. In this example, between one and ten behavior categories are observed per observation. All behavior categories are observed equally (e.g., when four behavior categories occur, they are all observed equally often). The figure demonstrates the effect of increasing numbers of observations of each behavior on the index values generated. Here, each behavior occurs at a different frequency (either once, twice, thrice, four, five, ten or one hundred times, during separate simulations). The figure shows that Simpson’s index values are not generated when only one behavior category occurs, and generally, higher index values are generated when behaviors are observed many times.

Practitioners should note, however, that there is one potential shortfall to the three Simpson’s indices. Unlike the Shannon–Wiener index, which runs on proportional data, Simpson’s indices run on actual, observed values. This means that different values are generated when the number of observations made are larger. For example, two behaviors may occur equally commonly in two observations, but a different value will be generated if, for example, the animal was out of sight for a period of time (see Figure 4). This should be considered carefully, especially in studies where animals are likely to be out of sight or observations may be of different lengths [30]. This said, the Simpson’s indices could be applied to studies where comparisons need to be made in behavioral diversity, for example between pre-enrichment and post-enrichment conditions. The advantage to these indices are the more clearly defined set of values in which diversity may fall.

### 4.4. Menhinick’s Index

Menhinick’s index is a very simple index that has been widely practiced in ecology [26]. The equation for Menhinick’s index (*R*_1_) is:

$$R_{1}=\frac{S}{\sqrt{N}}$$

Here, *S* refers to the number of behavior categories, and *N* denotes the total number of observations. Values vary, with a minimum value of 0 (large number of behavioral observations but only one behavior seen) and maximum values extending beyond 1 (lots of behaviors seen with only a few observations). The value of 1 essentially indicates that the number of behavioral observations is equivalent to the square root of the number of behaviors seen. Thus, a value of 1 can be generated if four observations are conducted and two behaviors are seen, or nine observations are conducted and three behaviors are seen.

Unlike the Shannon–Wiener index, Menhinick’s index is not sensitive to evenness. An observation where all behaviors are seen equally, and an observation where only one behavior dominates, would therefore produce the same Menhinick’s value, so long as both observations were of the same length and the number of behaviors seen was identical. This, therefore, is a potential limitation to the index, as behavioral richness features much more prominently.

**Figure 5:**
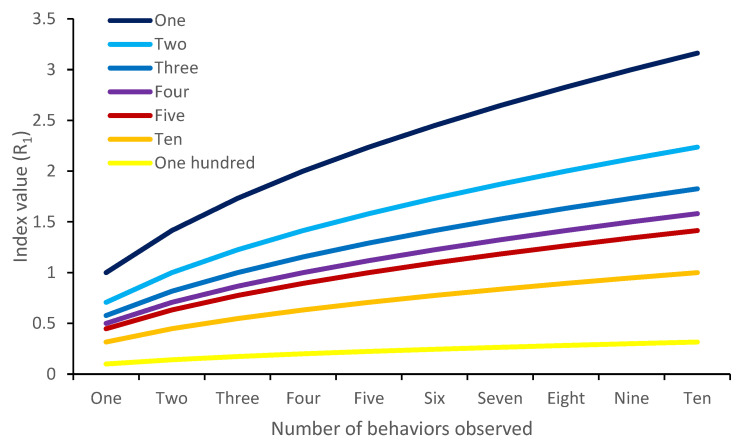
Menhinick’s index values when each behavior is seen equally at a rate of one, two, three, four, five, ten or one hundred times in an observation period.

The index, unlike the Shannon–Wiener index, is also much more sensitive to sample size (number of observations made). As a result, Menhinick’s index scores tend to become lower when more observations are undertaken (Figure 5).

### 4.5. Margalef Index

**Figure 6:**
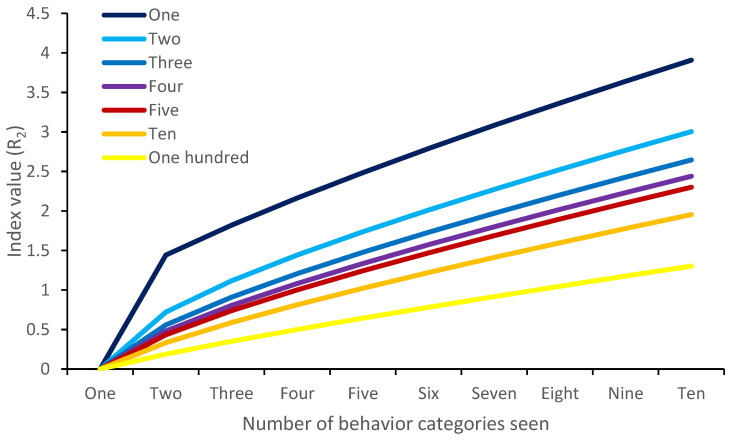
Margalef index values when each behavior is seen equally at a rate of one, two, three, four, five, ten or one hundred times in an observation period. The Margalef index does not take into account evenness of observations.

The Margalef diversity index (*R_2_*) is regularly applied in ecology [38] but does not feature in the animal behavior literature. The equation for Margalef’s index is:

$$R_{2}=\frac{S-1}{Ln\left(N\right)}$$

whereas *S* refers to the number of categories of behavior, and *N* denotes the total number of observations. Unlike Menhinick’s index, this index uses a natural logarithm to generate index values. Values of 0 indicate lower behavioral diversity, with higher values indicating a higher number of behavior categories observed. As per Menhinick’s index, these values are reduced by N, with higher numbers of observations resulting in lower behavior scores (Figure 6).

Because of the ease with which both the Menhinick’s and Margalef indices can be calculated, they could be applied to studies where a rough behavioral diversity value is required, for instance in initial assessments of the impact of enrichment introductions on different individuals. However, these two indices should be avoided in studies where evenness of behavior is important.

### 4.6. Chao

The Chao index is well applied in the field in microbiology, where there is a need to assess large numbers of microbial species, often with many unobserved taxa [39]. At current, Chao has not been applied to behavioral diversity research, yet there is considerable potential for this technique. Unlike other indices, Chao (sometimes referred to as *Chao*1) works by estimating the true number of species in an ecosystem or sample. The equation is as follows:

$$Chao1=Sobs+\frac{F_{1}\left(F_{1}-1\right)}{2\left(F_{2}+1\right)}$$

Here, *Sobs* refers to the number of species observed (or for behavioral diversity, the number of behavior categories observed). *F*_1_ refers to the number of behaviors where only a single incidence of the behavior was observed across the observation period (singleton), and *F*_2_ refers to the number of behavior categories that were seen only twice (doubletons). From these data, Chao generates an estimate value for the true number of behavior categories the animal may express.

**Figure 7:**
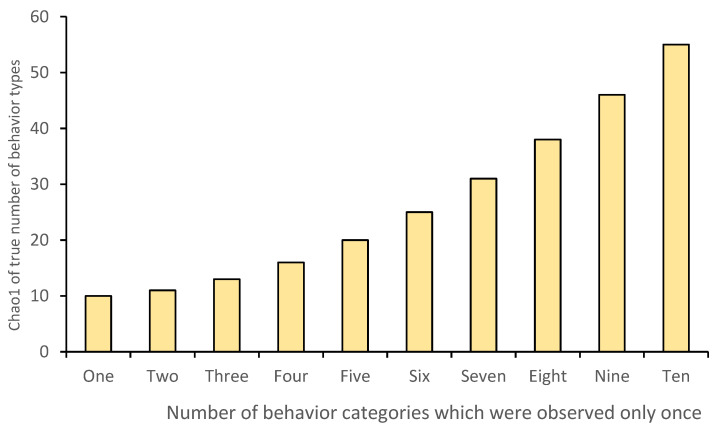
Simulated observations where ten categories of behavior are observed. The figure shows the effect on Chao1 values of an increasing number of observations where a behavior occurs only once in the observation period.

Chao index values are identical to the number of behaviors actually observed, unless single observations of a behavior category occur. The more single observations of behaviors that appear, the higher the estimates of the ‘true’ number of behaviors (Figure 7).

In terms of value to researchers, the Chao index has great potential as a tool for pilot studies. The index could be applied to estimate the ‘true’ number of behaviors that an animal may express. For example, a researcher might observe a previously unstudied species for a short observation period using a standardized observation technique (e.g., instantaneous sampling; see [15] for detailed descriptions of behavioral observation methods). The longer the observer is present, the greater the chances of seeing rare behaviors (e.g., courtship, social behavior, nesting). The Chao index provides an estimation of how many behaviors are yet to be discovered based on the rate of discovery.

### 4.7. Behavioral Variety Index

The Behavioral Variety index (BVI) features in some animal behavior literature (e.g., 20,21), and has some potential in investigating animal welfare. BVI requires a full ethogram of an animal its ‘natural’ state to determine the range of behaviors it may express [20,21]. These behaviors are then categorized into groups—such as social behavior. Following this, observations of the target population are commenced, and the presence or absence of each behavior category per animal is recorded. This is converted into a percentage *BVI* value per animal. For this index, low index values indicate poorer behavioral diversity. The equation is:

$$BVI=\frac{Ob}{Ex}\;\times \;100$$

where *Ob* refers to the number of categories of behavior observed in the study, and *Ex* refers to the number of behaviors expected (based on wild studies or published literature. There are some potential hazards, for instance where wild behaviors are not conducive to either captive comparisons or ideal captive welfare (e.g., anti-predatory or vigilance behavior). The index can, however, provide excellent comparisons between habitats and provide some informed feedback in opportunities for animals to express natural behaviors. Finally, it is also worth noting that this index does not take into account aspects of behavioral evenness; only presence or absence of behaviors are recorded.

## 5. Comparison of Indices

Overall, this study revealed that the majority of animal research papers that used the term ‘behavioral diversity’ made use of the Shannon–Wiener index [40,41]. The second most common method was behavioral richness; a simple measure of the number of behaviors observed. Many indices, such as Simpson’s and Menhinick’s indices, did not feature in the identified papers, whereas the Behavioral Variety Index featured only twice.

The Shannon–Wiener index is likely to be prevalent in the literature because it considers both behavioral richness and evenness [42,43]. It is likely that the index is used because it has been validated in previous research studies [10]. However, it should be noted that the index does not consider the number of observations conducted. Other techniques, such as the Simpson’s index, do not rely on proportional data and place slightly greater emphasis on observation number. The same is true for both Margalef and Menhinick’s indices. There is scope for researchers to extend the number of tools they use when investigating behavioral diversity (Table 2)

It is surprising that the second most common index of behavioral diversity was richness. While richness has value as a diversity indicator, it has several limitations [44]. Behavioral richness (number of behaviors seen) does not take into account evenness or the number of observations, or the behavior types that were recorded [45,46]. This leaves the values to be easily skewed in a real-world setting, such as in studies that observed animals over differing lengths of time or used different sampling interval lengths [47]. Furthermore, the lack of consideration of evenness is a greater limitation because rare behaviors are just as well presented in scores as are common behaviors.

The ability to estimate the true number of behaviors an animal may express is particularly novel, and achievable through the Chao index [48]. This could give researchers a more informed estimate as to how long they may need to observe their animals to identify all rare behaviors, such as reproductive behaviors. (Here, consideration should also be paid to the use of environmental variables or objects as cues for rare behaviors).

In future behavioral diversity studies, researchers could avoid some of the shortfalls associated with any single index by applying a combination of several indices. Such methods are commonly used in ecology and microbiology, where combinations in Simpson’s and/or Shannon–Wiener index, plus Chao, are commonplace [24,26].

### Taxonomic Differences

Not all taxonomic groups were equally well represented in behavioral diversity literature. For example, mammals were the subject species for 75% (84/112) of the identified literature [49,50]. Of these studies, the Orders of Carnivora [50,51,52,53,54,55,56,57], Cetacea [10,46] and primates [18], particularly chimpanzees (*Pan troglodytes)* [58,59,60,61] were the most commonly studied groups. While reptile [62,63,64], bird, fish [65] and invertebrate [66,67] species appeared in some studies, they were in the minority overall. No studies of behavioral diversity in amphibians were identified.

Similar trends in species bias have been identified in the animal literature, in which a few mammalian orders have been exceptionally well-studied, while and other taxa, such as reptiles, amphibians, and invertebrates, have not (e.g., [68]). Much of the wider zoo research is focused on behavior [69], so the prevalence of specific orders is likely reflective of wider zoo patterns. Use of behavioral diversity indices (and more widely behavioral studies) could help researchers to better understand the natural behavior and welfare of animals. For example, these studies could help researchers to identify whether behavioral diversity levels are similar between related animal species, and where adjustments to animal husbandry and welfare may be needed. Additionally, greater focus on amphibians, fish and vertebrates for behavioral diversity research would be beneficial. This could help to build the evidence base on which welfare and animal needs could be better understood. Increased application with farm animals, which are well represented in the research (e.g., [70]), would also be beneficial, as research could be used to understand and compare the behavior of a large number of individual animals in agricultural settings.

## 6. Conclusions

Other than a simple measure of the number of behaviors (i.e., behavioral richness), the most commonly used measure of behavioral diversity in the animal behavior literature was the Shannon–Wiener index. While a straightforward and valuable animal welfare tool, there remain many unused indices in the behavioral diversity toolkit. Use of further measures, such as the Chao and Simpson’s indices, could allow researchers to make more informed decisions regarding the length of their studies, the frequency of their observations, and the number of behaviors that have not yet been observed. Diversification of index use, therefore, has great potential in providing a more holistic understanding of animal welfare for scientists. While taking heed of the limitations of the diversity indices, researchers should develop their use of these tools for a wider array of study subjects, taxa, and settings.

## Figures and Tables

**Table 1:** Number of species studied using behavioral diversity measures in published studies.

Taxon	Shannon–Wiener	Richness	Behavioral Variety	Total
Amphibians	0	0	0	0
Birds	1	11	1	13
Fish	3	2	0	5
Invertebrates	0	2	0	2
Mammals	67	16	1	84
Reptiles	8	0	0	8
Total	79	31	2	112

**Table 2:** Comparison of indices and their potential application to behavioral diversity study.

	Does the Index Take Into Account:			
	Number of Behaviors	Evenness of Behaviors	Number of Observations	Notes
Shannon–Wiener	Yes	Yes	No	Based on proportion data.
Simpson’s	Yes	Yes	Yes	Not based on proportions, considers evenness.
Menhinick’s	Yes	No	Yes	Evenness not considered by this index.
Margalef	Yes	No	Yes	Evenness not considered by the index.
Chao	Yes	No	Yes	Used for estimating the number of missing behavior categories.
Behavioral Variety Index	Yes	No	No	Developed for comparing captive animal behavior against wild-type behavior.

## Data Availability

Data can be generated using the spreadsheets provided as Supplementary Material.

## Supplementary Materials

The following supporting information can be downloaded at: https://www.mdpi.com/article/10.3390/ani12212984/s1, Behavioural Diversity toolkit spreadsheet for calculations for indices.

## Appendix A

**Table A1:** Glossary of key terms.

Term	Definition
Behavioral diversity	Any index used to assess the quantify the evenness and diversity of behaviors that an animal expresses.
Behavioural evenness	The variance in quantity of behavior between behavior types. For example, an observation where all behaviors are seen equally frequently has a higher evenness score than an observation when only one behavior is seen frequently, and all others are seen rarely.
Behavioral richness	The number of behaviors an animal shows during a study. For example, an animal that engages in eating, resting and climbing has a behavioral richness of 3.
Behavior category	A behavior that can be defined in an ethogram (for example, foraging). For the purpose of this paper, the term ‚behavior type‘ used in other research is synonymous. The number of behavior categories identified in a behavioral diversity study is referred to as behavioral richness.
Observation	A block of time during which animals are observed (e.g., 1 h observation). A study is made up of a series of observations.
